# Society of Veterinary Graduates of Wisconsin

**Published:** 1901-08

**Authors:** W. G. Clark

**Affiliations:** Secretary


### SOCIETY OF VETERINARY GRADUATES OF WISCONSIN.

THE annual meeting of the Society of Veterinary Graduates was called to order in the capitol building, Madison, Wis., on February 5, 1901, at 2.30 P.M., by the President, Dr. A. H. Hartwig.

On roll-call there were present:

Drs. H. A. Arpki, S. Beattie, W. G. Clark, B. L. Clarke, H. P. Clute, S. J. Collins, R. E. Cochrane, C. M. Crane, E. Evans, H. F. Eckert, A. H. Hartwig, R. S. Heer, G. Ed. Leech, E. A. McCullough, E. H. Newton, A. J. Nelson, J. M. O'Reilley, J. F. Roub, D. Roberts, E. D. Roberts, Charles Schmitt, E. R. Flack, S. S. Snyder, L. A. Wright, and the following visitors: Farmer Miles, Charleston, Ill.; J. M. Armstrong, Richland Centre; A. L. Fosse, Deerfield; L. M. Jargo, Jefferson; J. S. Phieffer, Franklin; George E. Allen, Fort Atkinson; William J. Malone, Mt. Horeb.

The minutes of the semi-annual meeting were read and approved. On motion, the Society adjourned, subject to call, to the meeting of the State Agricultural Society, to listen to Dr. H. P. Clute's paper, subject, "Tuberculosis."

After this the meeting was again called to order. The Secretary's report of accounts was read and accepted. The Treasurer's report was read and accepted. The Secretary reported that Dr. C. A. Woodford, of Rio, had paid his dues in full and requested to withdraw, as he had retired from active practice, and recommended that he be placed on the honorary list.

It was moved and seconded that Dr. Woodford's name be placed on the list of honorary members, and that the Secretary so advise Dr. Woodford; carried.

The following applications for membership were received: Andrew L. Fosse, Ontario Veterinary College, Deerfield; Joseph T. Hernshein, University of Pennsylvania, Kenosha; J. M. Armstrong, Ontario Veterinary College, Richland Centre; L. M. Jargo, Ontario Veterinary College, Jefferson; W. S. Powell, McKillip Veterinary College, Marshfield; Joseph S. Phieffer, Chicago Veterinary College, Franklin; William J. Malone, Chicago Veterinary College, Mt. Horeb. The censors reporting favorably, it was moved and seconded that the candidates be elected by acclamation; carried. The above-named gentlemen were declared duly elected to membership.

The Secretary reported that Dr. Charles Kochne had met with an accident with a corn-husking machine, losing his right hand, except his thumb, and recommended that he be placed on the list of honorary members. On motion, Dr. Charles Kochne was elected an honorary member of the Society.

The Secretary read the report of the Committee on Collections and several letters received from members in arrears. Dr. Arpki recommended that those several years in arrears be dropped from the list of members. Dr. Leech thought that back dues could not be collected under the present By-laws, and recommended an amendment to Section 12, and that discussion be postponed until the evening session. On motion, this carried.

Dr. H. P. Clute, chairman of the Committee on Legislation, reported that the committee had held a meeting in Milwaukee with a committee from the State Board of Agriculture and the Experiment Station in regard to the establishment of a Live-stock Sanitary Board and in regard to proposed changes in present State veterinary laws. Discussed by Drs. E. D. Roberts and D. Leech. On motion, the report was accepted.

Dr. Beattie, secretary of the Committee on Prosecution of Illegal Practitioners, stated that several had retired from business as a result of the work of the committee and Dr. Hartwig. Discussed by Drs. Leech, Clute, Collins, and O'Reilley.

Dr. Leech requested the privilege to revert to new business. There being no objection the request was granted, and Dr. Leech requested the Secretary to read an extract from the Chicago *Horseman* of January 29, 1901, in regard to the question of giving rank to veterinary surgeons in the United States Army, and asked that the communication be officially answered. After discussion, Dr. D. Roberts moved that a press committee be appointed to answer this communication and any other matter that might be necessary to be placed before the public. Seconded by Dr. Leech ; carried.

Moved to adjourn until 7.30 P.M. ; carried.

*Evening Session.*—The meeting was called to order at 7.30 P.M. by the President.

The subject of collections from those in arrears was taken up, and the Secretary was instructed to correspond with those more than four years in arrears and report at the semi-annual meeting. Moved and seconded that those who refuse to pay be suspended at the semi-annual meeting for non-payment of dues ; carried. Moved by Dr. Leech, and seconded by Dr. Crane, that Section 12 be amended to read, "That no member can withdraw from this Society unless his dues be paid in full ;" carried.

The President appointed as a Press Committee, Drs. G. Ed. Leech, Milwaukee ; R. E. Cochrane, Milwaukee ; C. Evans, Racine.

*Reading of Essays and Communications:* The venerable Farmer Miles, of Charleston, Ill., was introduced by the President, and proceeded to tell in his inimitable manner how he came to commence the practice of the specialty which has made his name familiar throughout the world as a castrator of cryptorchids. In describing the operation he divides them into six classes, as follows : No. 1. "Flankers." No. 2. Testicle outside abdominal cavity, but so near the inguinal ring that it cannot be felt. No. 3. The testicle and appendages inside the abdomen. This class comprises three out of five of straight cryptorchids. No. 4. Tunic and globus minor down, but testicle within the abdominal cavity. No. 5. Once straight No. 3, but from some cause an internal hydrocele formed which sometimes contains two or three pints of fluid with the testicle attached to some part of the sac. No. 6. Once No. 5, but nature makes an effort to reduce the hydrocele, and a hard cheesy mass is formed having the consistency of a soft brick.

The most important part to learn is to study to go slow. In a straight colt six to eight inches of the spermatic cord should be removed with the écraseur, and there will be no trouble with schirrous cords. A large external opening should be made to provide free drainage. Open this twice daily by inserting the fingers for five days, then allow it to heal.

He then proceeded to describe in detail the several operations on the different classes of ridglings. After this he related numerous anecdotes regarding his personal appearance. He has a vast fund of quaint humor, and is as interesting as a romance, and it was his aim to make plain all the little secrets of his art that he has prized so highly, and now he is retiring at the age of seventy-five years, and is anxious to benefit the members of the veterinary profession as far as may lie in his power. On motion, a vote of thanks was tendered Farmer Miles for his communication.

It was moved and seconded that Farmer Miles be elected an honorary member of the Society; carried. Farmer Miles was declared elected an honorary member.

Dr. H. F. Eckert read a paper on "Tænia Expansa in the Sheep."

Discussed by Drs. Schmitt, Crane, Hartwig, Wright, D. Roberts, Heer, and Leech. On motion, the essayist was excused.

Dr. Charles Schmitt read a paper on "Intussusception and Surgical Treatment," describing a case occurring in his practice and the operation by means of an appliance resembling the Murphy button. Discussed by Drs. Hartwig, Heer, Leech, and E. D. Roberts.

Dr. E. D. Roberts moved that the Society indorse Dr. H. P. Clute for a position in the Veterinary Corps of the United States Army under the Army Reorganization bill, and the Secretary be instructed to draft a resolution to that effect. This was seconded and carried unanimously.

*Election of Officers.* The following officers were elected for the ensuing year: President, C. Evans, Racine; Vice-President, J. F. Roub, Monroe; Secretary, W. G. Clark, Marinette; Treasurer, S. S. Snyder, Cedarburg. Censors: H. P. Clute, Marinette; B. L. Clarke, Monticello, and R. S. Heer, Platteville.

On motion, a vote of thanks was tendered Drs. Hartwig and W. G. Clark for their labors for the Society during the past year; carried.

On motion, a vote of thanks was tendered Dr. Beattie for his services in providing for the accommodation of the members at the hotels and arrangements made for the meeting.

After discussion it was decided to meet in Milwaukee during the week of the State Fair in September.

On motion, Drs. D. Roberts, Leech, Onnond, and Cochrane were appointed a Committee on Arrangements. On motion, the Society adjourned.

*Clinics.*

The members met in the stock-judging room at the Agricultural Experiment Station, February 6th, at 8.50 A.M.

Farmer Miles, assisted by Drs. Schmitt, Wright, and Collins, spayed two heifers and castrated two cryptorchids.

Dr. H. P. Clute, assisted by Drs. Beattie and Hartwig, performed arytenoidotherapy on a driver, belonging to Dr. Beattie, afflicted with roaring.

Dr. Beattie reports after a month that the result was successful.

W. G. Clark,
Secretary.

